# Supplementary material for: Splice-Junction-Based Mapping of Alternative Isoforms in the Human Proteome
Source: Cell Rep. Author manuscript; Available in PMC 2020 Jan 15. (PMC6961840; doi:10.1016/j.celrep.2019.11.026)
Supplement: 2 [file NIHMS1546469-supplement-2.zip › DF1/PXD009737/TestisTrypsin/TestisTrypsin_1_ZFPM1_ELEGQEPEPRPTEEEPGSPWSGPEPSPDPAAGGR.pdf]

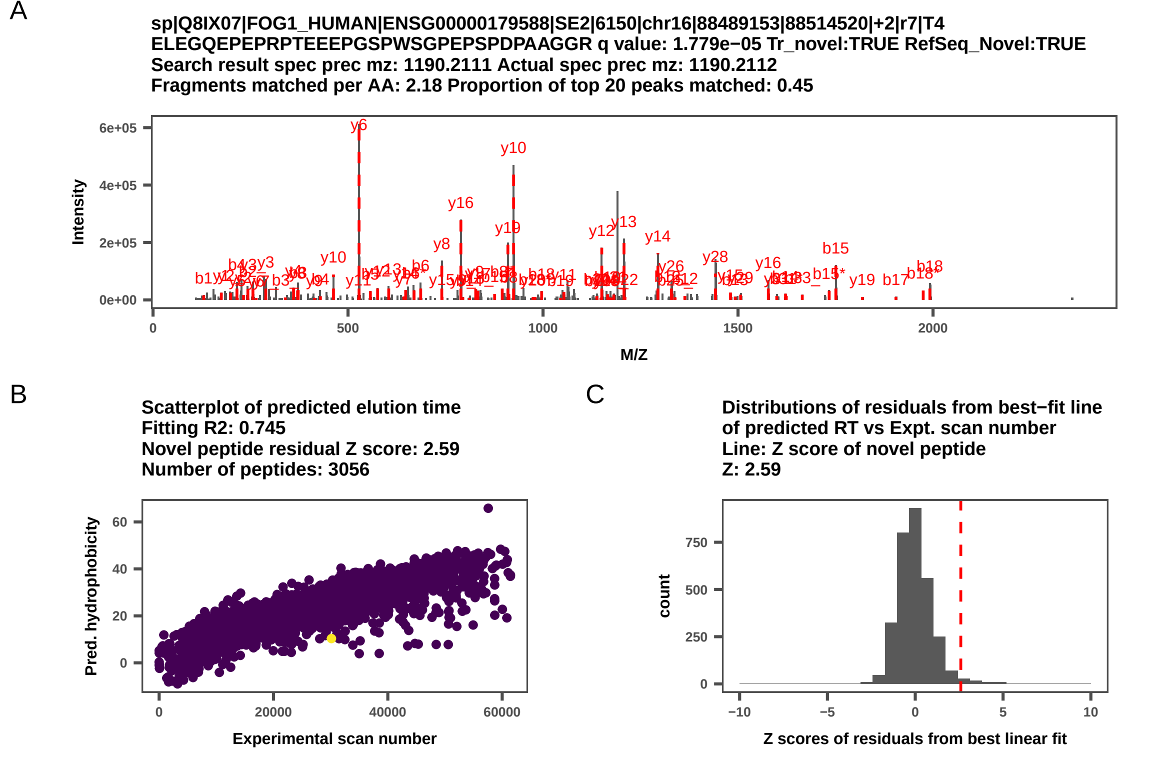
A
sp|Q8IX07|FOG1_HUMAN|ENSG00000179588|SE2|6150|chr16|88489153|88514520|+2|r7|T4
ELEGQEPEPRPTEEEPGSPWSGPEPSPDPAAGGR q value: 1.779e−05 Tr_novel:TRUE RefSeq_Novel:TRUE
Search result spec prec mz: 1190.2111 Actual spec prec mz: 1190.2112
Fragments matched per AA: 2.18 Proportion of top 20 peaks matched: 0.45
Intensity
6e+05
4e+05
2e+05
0e+00
0
500
1000
1500
2000
M/Z
y6
y10
y16
y19
y8
y12
y13
y14
y28
y16
b15
b18
y19
b17
B
Scatterplot of predicted elution time
Fitting R2: 0.745
Novel peptide residual Z score: 2.59
Number of peptides: 3056
Pred. hydrophobicity
60
40
20
0
0
20000
40000
60000
Experimental scan number
C
Distributions of residuals from best−fit line
of predicted RT vs Expt. scan number
Line: Z score of novel peptide
Z: 2.59
count
750
500
250
0
−10
−5
0
5
10
Z scores of residuals from best linear fit
